# Supplementary material for: From sequence to enzyme mechanism using multi-label machine learning
Source: BMC Bioinformatics. 2014 May 19;15:150. doi: 10.1186/1471-2105-15-150 (PMC4229970; doi:10.1186/1471-2105-15-150)
Supplement: Additional file 2 — Java code of ml2db. Additional file ml2db_code.tar.gz contains the Java source code to run the multi-label machine learning experiments and save the results to database. The code’s Javadoc is included. [file 1471-2105-15-150-S2.zip › additional file 2/ml2db/utils/doc/edu/cornell/lassp/houle/RngPack/RandomApp.html]

RandomApp


JavaScript is disabled on your browser.


- Overview
- Package
- Class
- Use
- Tree
- Deprecated
- Index
- Help

- Prev Class
- Next Class

- Frames
- No Frames

- All Classes

- Summary:
- Nested |
- Field |
- Constr |
- Method

- Detail:
- Field |
- Constr |
- Method


edu.cornell.lassp.houle.RngPack

## Class RandomApp

- java.lang.Object
- - edu.cornell.lassp.houle.RngPack.RandomApp

- ---

    

  ```
  public class RandomApp
  extends java.lang.Object
  ```

  RandomApp is a simple application that demonstrates the use of RngPack.
  RandomApp generates random numbers and writes them to the standard output.
  This is very useful on Unix systems since the output can be piped to another
  application. See RandomApp documentation for
  how to run it.

  Source code
   is available.

  Version:
  :   1.0

  Author:
  :   Paul Houle  (E-mail:
      houle@msc.cornell.edu)

  See Also:
  :   `RandomJava`,
      `RandomShuffle`

- - ### Constructor Summary

    Constructors

    Constructor and Description

    `RandomApp()`
  - ### Method Summary

    Methods

    | Modifier and Type | Method and Description |
    | --- | --- |
    | `static void` | `main(java.lang.String[] args)` |

    - ### Methods inherited from class java.lang.Object

      `equals, getClass, hashCode, notify, notifyAll, toString, wait, wait, wait`

- - ### Constructor Detail


    - #### RandomApp

      ```
      public RandomApp()
      ```
  - ### Method Detail


    - #### main

      ```
      public static void main(java.lang.String[] args)
      ```


- Overview
- Package
- Class
- Use
- Tree
- Deprecated
- Index
- Help

- Prev Class
- Next Class

- Frames
- No Frames

- All Classes

- Summary:
- Nested |
- Field |
- Constr |
- Method

- Detail:
- Field |
- Constr |
- Method
